# Identifying the gene responsible for non‐photochemical quenching reversal in *Phaeodactylum tricornutum*

**DOI:** 10.1111/tpj.17104

**Published:** 2024-10-30

**Authors:** Maxwell A. Ware, Andrew J. Paton, Yu Bai, Tessema Kassaw, Martin Lohr, Graham Peers

**Affiliations:** ^1^ Department of Biology Colorado State University Fort Collins Colorado 80523 USA; ^2^ Fachbereich Physik Freie Universität Berlin Berlin 14195 Germany; ^3^ Institut für Molekulare Physiologie Johannes Gutenberg‐University Mainz 55099 Germany

**Keywords:** nonphotochemical quenching, diatom, photosynthesis, diatoxanthin, diadinoxanthin, xanthophyll cycle

## Abstract

Algae such as diatoms and haptophytes have distinct photosynthetic pigments from plants, including a novel set of carotenoids. This includes a primary xanthophyll cycle comprised of diadinoxanthin and its de‐epoxidation product diatoxanthin that enables the switch between light harvesting and non‐photochemical quenching (NPQ)‐mediated dissipation of light energy. The enzyme responsible for the reversal of this cycle was previously unknown. Here, we identified zeaxanthin epoxidase 3 (ZEP3) from *Phaeodactylum tricornutum* as the candidate diatoxanthin epoxidase. Knocking out the *ZEP3* gene caused a loss of rapidly reversible NPQ following saturating light exposure. This correlated with the maintenance of high concentrations of diatoxanthin during recovery in low light. Xanthophyll cycling and NPQ relaxation were restored via complementation of the wild‐type ZEP3 gene. The *zep3* knockout strains showed reduced photosynthetic rates at higher light fluxes and reduced specific growth rate in variable light regimes, likely due to the mutant strains becoming locked in a light energy dissipation state. We were able to toggle the level of NPQ capacity in a time and dose dependent manner by placing the ZEP3 gene under the control of a β‐estradiol inducible promoter. Identification of this gene provides a deeper understanding of the diversification of photosynthetic control in algae compared to plants and suggests a potential target to improve the productivity of industrial‐scale cultures.

## Significance Statement

This work reveals the identify of the enzyme responsible for the deactivation of the xanthophyll cycle and photoprotection in diatoms.

## INTRODUCTION

There are a diverse set of pigments used by plants and algae for photosynthesis and for photoprotection. Researchers focused on vascular plants are familiar with chlorophylls *a + b*, which are used for light energy capture (Kunugi et al., 2016). Conversely, the carotenoid pigments of the xanthophyll cycle (violaxanthin, antheraxanthin and zeaxanthin; VAZ) are known to play an important role in regulating the thermal dissipation of excess light energy through non‐photochemical quenching (NPQ, Jahns et al., 2009). The enzymes violaxanthin de‐epoxidase (VDE) and zeaxanthin epoxidase (ZEP) contribute to toggling the light‐harvesting antennae between a light‐harvesting state and energy‐dissipation state (Bugos & Yamamoto, 1996; Jahns et al., 2009; Marin et al., 1996). VDE removes the epoxide group from the ionone rings of violaxanthin to yield zeaxanthin which promotes NPQ. ZEP converts zeaxanthin back to violaxanthin, thereby re‐establishing efficient light utilization.

Many algae, examples of which include diatoms and haptophytes, contain a different set of photosynthetic pigments from plants. The brown color of these algae is primarily due to the presence of chlorophylls *a + c* and the carotenoid fucoxanthin which participate in light harvesting (Büchel, 2020). Furthermore, the xanthophyll cycle of these algae utilize diadinoxanthin and diatoxanthin (Ddx:Dtx), which are absent from the green lineage of algae and plants (Bai et al., 2022; Takaichi, 2011). Under certain conditions, algae with the Ddx:Dtx cycle may also accumulate the pigments of the VAZ cycle, but probably not to a significant extent under most natural conditions (Lohr & Wilhelm, 1999).

NPQ is the major photoprotective mechanism in diatoms. Indeed, the rapidly inducible component of NPQ, qE, can dissipate 50% of absorbed photons as heat (Giovagnetti et al., 2014). The de‐/epoxidation cycle works as follows: Ddx is the xanthophyll correlated with light harvesting under light limited conditions, and a high luminal pH. During high light (HL) induced low luminal pH, Ddx is de‐epoxidated to Dtx. This promotes NPQ similar to zeaxanthin in the VAZ cycle (Arsalane et al., 1994). Gene silencing experiments suggested that the VDE gene is a key component for the conversion of Ddx to Dtx in diatoms (Lavaud et al., 2012). However, the reverse reaction has not been resolved.

Reverse genetics experiments facilitate the discovery of biosynthetic pathways associated with light harvesting and NPQ‐related pigments in diatoms and related algae. For instance, there has also been increased interest in uncovering the biosynthesis pathway of fucoxanthin. This pigment is an asymmetrical carotenoid containing unusual chemical features such as an allenic bond and a keto group in its polyene moiety. In diatoms and haptophytes, fucoxanthin biosynthesis is facilitated by a series of gene duplications and neofunctionalizations that occurred during/post‐secondary endosymbiosis. It has been determined that paralogues of VDE and ZEP contribute to fucoxanthin biosynthesis. This was surprising as VDE and ZEP play roles in NPQ related xanthophyll cycling in green algae and plants, as described above. Violaxanthin De‐Epoxidase‐Like 1 (VDL1) catalyzes the tautomerization of violaxanthin to neoxanthin (Dautermann et al., 2020). VDL2 performs the tautomerization of diadinoxanthin to allenoxanthin (Bai et al., 2022). Meanwhile, the epoxidation of haptoxanthin to phaneroxanthin, the penultimate step of fucoxanthin biosynthesis, is catalyzed by ZEP1 (Bai et al., 2022).

The genome of the model diatom *Phaeodactylum tricornutum* encodes two additional *ZEP* genes (ZEP2 and ZEP3, Bai et al., 2022; Coesel et al., 2008). We hypothesized that ZEP3 may be playing a role in the photoprotective Ddx:Dtx cycle as its expression has been found to be co‐regulated with that of VDE (Nymark et al., 2013). Additionally, these two genes appear to be driven by a common bi‐directional promoter that is conserved throughout diatom genomes. This suggested to us a shared function in regulating NPQ. Here we show that insertional knockout of the ZEP3 gene results in a reduced variable NPQ capacity due to the constitutive accumulation of diatoxanthin and reduced growth rates in variable light environments compared to WT strains.

## RESULTS

### Knockout of PtZEP3

**Figure 1:**
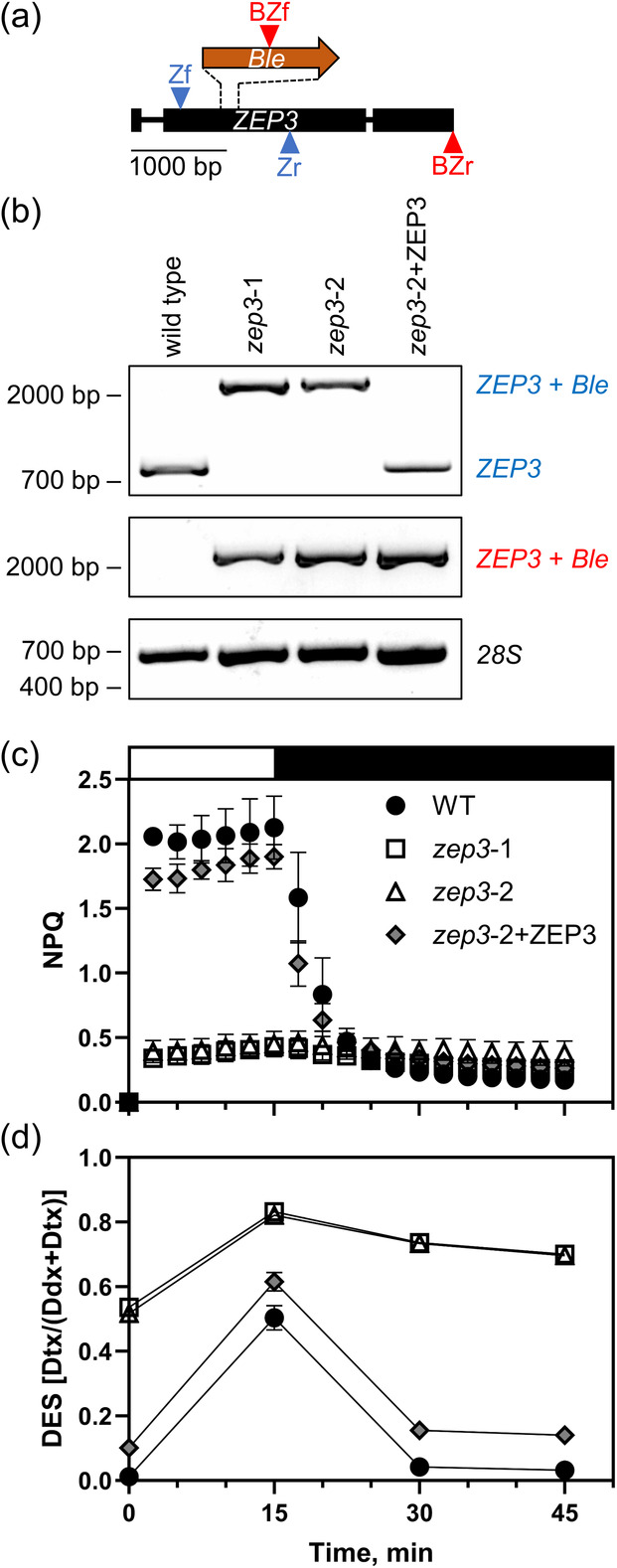
ZEP3 gene knockout design, verification of mutations, quantification of NPQ capacity and xanthophyll cycling in WT, mutant and complemented strains. (a) Schematic of *PtZEP3* gene and insertion of bleomycin resistance gene (*Ble*) for knockout. (b) Binding sites for guide RNAs are within the dashed lines, allowing for CRISPR‐mediated cutting and subsequent recombination of the *Ble* gene at the site. Primer binding sites are indicated by arrowheads, with forward primers above the genes and reverse primers below the genes. There is a primer pair for *ZEP3* amplification (Zf, Zr) and a primer pair for ZEP3+Ble amplification (BZf, BZr). Gel electrophoresis results for WT, two *zep3* mutants, and one *ZEP3* complemented strain. (c, d) Labels on the right show expected band positions for different strains, with the colored labels corresponding to the primer colors in (A) and a 28S amplification with a black label as a control. WT and complemented strains show a normal *ZEP3* band for the Zf/Zr amplification while mutants have a *ZEP3*+Ble band. Mutant and complemented strains show a *ZEP3*+Ble band for the BZf/BZr amplification while WT has no band. Relevant base pair migration positions from a Thermo Scientific 1 kb Plus DNA ladder are given on the left. B Cells were exposed to 15 min of high light (2000 μmol photons m^−2^ sec^−1^, white bar) and 30 min of low light (75 μmol photons m^−2^ sec^−1^, black bar). NPQ dynamics were measured with a DUAL‐PAM fluorometer (c) and DES was estimated via HPLC analyses (d). Dynamics were observed for Phaeodactylum WT, two *zep3* mutant strains, and one *ZEP3* complemented strain (*zep3*‐2 mutant complemented with native *ZEP3* gene, *zep3*‐2+ZEP3). Cells were grown in sinusoidal light and were collected an hour post‐dawn. Points are averages with error bars representing one standard deviation of biological replicates (*n* = 3).

We hypothesized that the *Phaeodactylum* gene annotated as zeaxanthin epoxidase 3 (*PtZEP3*, Phatr3_J10970) was the diatoxanthin epoxidase. We targeted a knockout of this gene using CRISPR‐Cas9 and homology directed repair‐mediated insertion of a bleomycin resistance cassette. We used two guide RNAs targeted to the 3′ end of exon 2 in the gene sequence (Figure 1a). This approach resulted in the generation of several homozygous *zep3* knockouts that grew on antibiotic selection. *ZEP3* gene disruptions via gene insertion were verified via PCR amplification of the *ZEP3* gene and its increased size compared to the WT (Figure 1b). Primers directed toward the *ble* antibiotic resistance gene and the 3′ end of the WT gene verified the disruption of *ZEP3* in our KO and complemented strains, with no product in the WT. Complementation of a mutant with the native gene sequence restored the WT band, which has been observed previously for this methodology (Figure 1, Bai et al., 2022).

### ZEP3 is necessary for rapidly reversible NPQ capacity and diatoxanthin epoxidation

WT, *zep3* KO strains, and complemented strains were cultivated in a 12 h light:12 h dark sinusoidal light (SL) regime. We first tested their NPQ capacity within an hour after dawn. At this time the light flux was approximately 500 μmol photons m^−2^ sec^−1^. The cultures were sampled and then left in low light (LL) for 1 h to allow for the relaxation of any induced NPQ and maximal relaxation of the xanthophyll cycle. When we then measured the NPQ capacity of WT, we found high NPQ capacity was rapidly induced with a saturating light intensity of 2000 μmol photons m^−2^ sec^−1^. NPQ then relaxed within 30 min of LL (Figure 1c). In contrast, there was very little induced NPQ observed in both *zep3* mutants and we observed little change throughout the LL relaxation phase (Figure 1c).

The de‐epoxidation state (DES) of a photosynthetic system refers to the proportion of xanthophyll cycle pigments which are de‐epoxidated at a given time. In WT diatoms, this is strongly positively correlated with induced NPQ (Lavaud et al., 2002) and is calculated as the amount of Dtx divided by the total amount of Ddx and Dtx. We therefore also measured the DES of these strains in a parallel NPQ induction experiment, with samples pulled from cultures at the same time as described immediately above. WT started off with a near‐zero DES in LL, followed by a large increase within 15 min of saturating light (Figure 1d). The DES returned to near‐zero between 15 and 30 min of LL relaxation (Figure 1d). This suggests a functioning diatoxanthin epoxidase in these conditions. Contrastingly, both mutants began the experiment with a high DES, approximately equal to the peak DES of wild type. The DES signal increased during saturating light and remained high during the LL relaxation (Figure 1d). This suggests a non‐functional diatoxanthin epoxidase in the *zep3* mutants. The ZEP3‐complemented line exhibited NPQ dynamics comparable to WT and a DES that also was induced and relaxed along with WT, although at a slightly higher baseline than WT (Figure 1d). Additional tested ZEP3‐complemented lines showed restored inducible NPQ capacity at variable peak levels compared to wild type (Figure S1).

**Figure 2:**
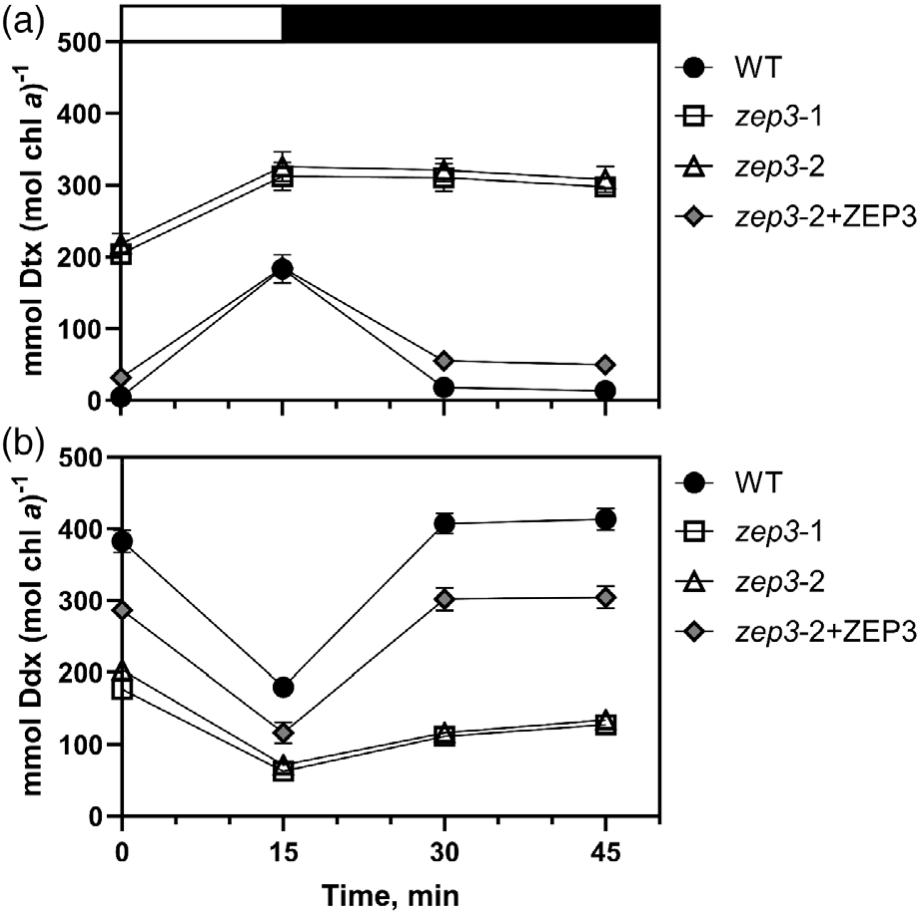
Diadinoxanthin (Ddx) and diatoxanthin (Dtx) dynamics of *Phaeodactylum* cultures during an non‐photochemical quenching (NPQ) induction experiment. Cells were exposed to 15 min of high light (2000 μmol photons m^−2^ sec^−1^, white bar) and 30 min of low light (75 μmol photons m^−2^ sec^−1^, black bar). Concentrations of Ddx (a) and Dtx (b) were measured via HPLC analyses and normalized to total chlorophyll *a*. Dynamics were observed for *Phaeodactylum* WT, two *zep3* mutant strains, and one *ZEP3* complemented strain. Points are averages with error bars representing one standard deviation of biological replicates (*n* = 3). Error bars may be smaller than symbols in some cases.

To further inspect the DES dynamics discussed above, we looked at the changing concentrations of diatoxanthin and diadinoxanthin individually. In WT, the epoxidated xanthophyll decreased from an average 383 to 180 mmol diadinoxanthin mol chl *a* ^−1^ over the course of the saturating light period, whereas the de‐epoxidated xanthophyll increased from 5 to 183 mmol diatoxanthin mol chl *a* ^−1^ in this time frame (Figure 2). This was mirrored by the relative changes at the end of the relaxation period, with a decrease of diatoxanthin concomitant with an increase of diadinoxanthin. In *zep3* lines, we observed a pattern that showed elevated diatoxanthin at the beginning of the experiments, conversion of diadinoxanthin to diatoxanthin, and then little change in epoxidation during the LL phase (Figure 2b). This further supports the role for the *ZEP3* gene being required for the epoxidation reaction correlating with the reversal of NPQ in LL. Notably, no violaxanthin, antheraxanthin, and zeaxanthin were observed in our experiments (Figure S2). These are the constituent pigments of the VAZ cycle. We propose the lower measured NPQ capacity of the mutants is due to the constitutive presence of Dtx, even after extensive dark acclimation, and that this causes NPQ to be locked “on”.

In a separate series of experiments, we grew the WT and KO strains in constant LL (60 μmol photons m^−2^ sec^−1^). We rationalized that the cells would not be exposed to saturating light intensities before measurement (Lavaud et al., 2002) and therefore we could expect the WT and KO strains to not be photoinhibited at the start of our assays. NPQ capacity assays of these LL acclimated cultures mutants showed rapid induction of NPQ in the WT and *zep3* strains. Again, little to no reversal of NPQ was observed in the mutant strains compared to the WT (Figure S3). These results show that the *zep3* mutants are able to induce NPQ if they are grown in LL. In these conditions, maximum quantum yield of PSII for WT and the two *zep3 s*trains were 0.692, 0.580 and 0.632, respectively (Figure S3d). This suggests that LL grown *zep3* strains show some constitutive quenching even after dark acclimation. The NPQ_(t)_ parameter permits the comparison of NPQ capacity when samples show constitutive quenching, photoinhibition, or when a dark acclimation period is not enough to completely regenerate maximal *F*_m_ (Tietz et al., 2017). Similar maximal values of NPQ_(t)_ between the mutants and WT (Figure S3e) suggest that the mutants do show constitutive quenching following dark acclimation, presumably due to the accumulation of Dtx.

### Growth of *zep3* is reduced in variable light regimes

**Figure 3:**
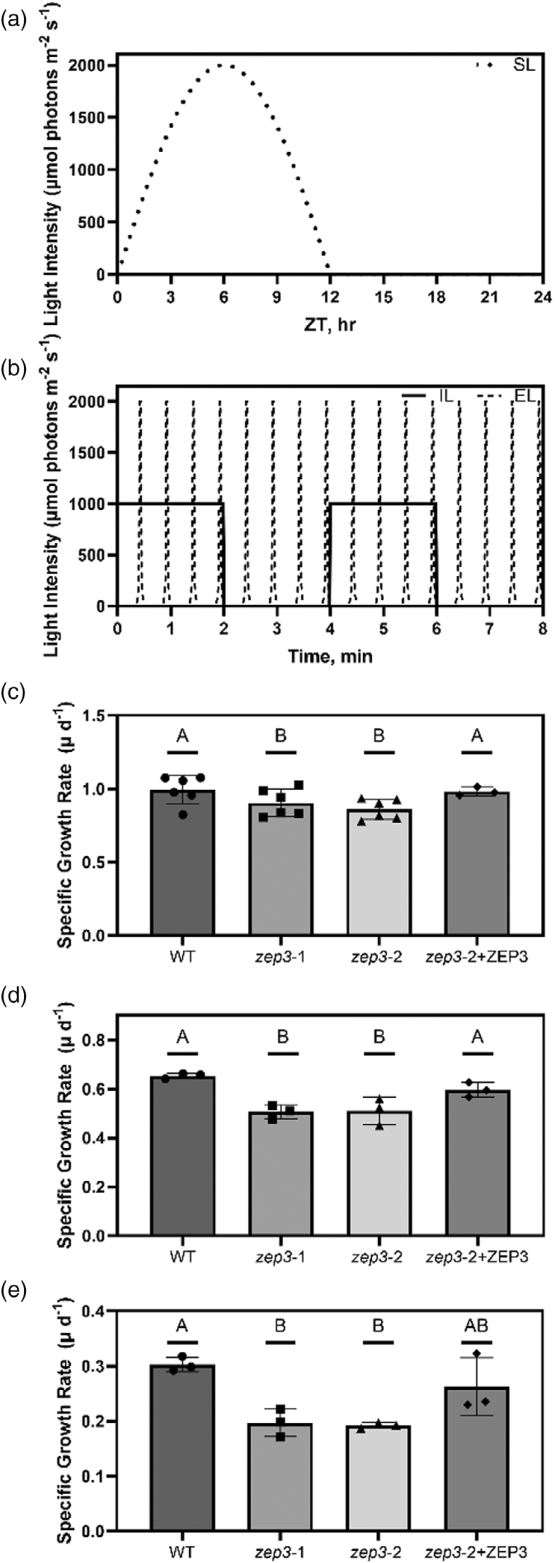
Maximal specific growth rates of *Phaeodactylum* cultures during different 12 h D:N variable light regimes. (a) Schematic of light regimes utilized in this experiment on a 24 h time scale, namely sinusoidal light (SL). (b) Schematic of the intermittent light (IL) and estuary light (EL) regimes on a time scale of several minutes during the day to visualize the finer resolution of light dynamics. (c–e) Specific growth rates of cultures grown in SL (c), IL (d), and EL (e) regimes. Growth rates were observed for *Phaeodactylum* WT, two *zep3* mutant strains, and one ZEP3 complemented strain. Bars are averages with points from individual replicate cultures shown and error bars representing one standard deviation (*n* = 6 or 3). Letters represent statistically different groups tested via a repeated measures one‐way analysis of variance (RM‐ANOVA) with a Tukey's HSD test.

Mutant plants and algae with an inability to regulate NPQ have reduced fitness in excess light and natural light regimes (e.g. Külheim et al., 2002; Peers et al., 2009). So, we next investigated the role of ZEP3 on fitness of *Phaeodactylum* in several different growth conditions. We used five light regimes, three of which were 12 h day:12 h night regimes with variable light during the day. SL increases from 0 to 2000 μmol photons m^−2^ sec^−1^ and then back to darkness in a 12‐h sinusoidal pattern before night (Figure 3a). Intermittent light (IL) comprised repeated intervals of 2 min of darkness and 2 min of 1000 μmol photons m^−2^ sec^−1^ light for 12 h before night (Figure 3b). Estuary light (EL) consisted of 8 sec spikes from 0 to 2000 μmol photons m^−2^ sec^−1^ and back every 30 sec for 12 h (Figure 3b), modeled from conditions a single cell would experience in a rapidly mixing estuary. In all these light conditions, both *zep3* strains demonstrated lower specific growth rate compared to WT, whereas the ZEP3‐complemented strain recovers growth rates to near wild‐type levels (Figure 3c–e). We also deployed two constant 24 h light regimes, low (LL) and high (HL) light at 60 and 450 μmol photons m^−2^ sec^−1^, respectively (Figure S4a). In these light regimes, *zep3 strains* did not grow slower than wild type (Figure S4b,c). This illustrates the importance of NPQ relaxation for efficient growth in variable light.

### Photosynthesis and NPQ of *zep3* are diminished at low and moderate light intensities

**Figure 4:**
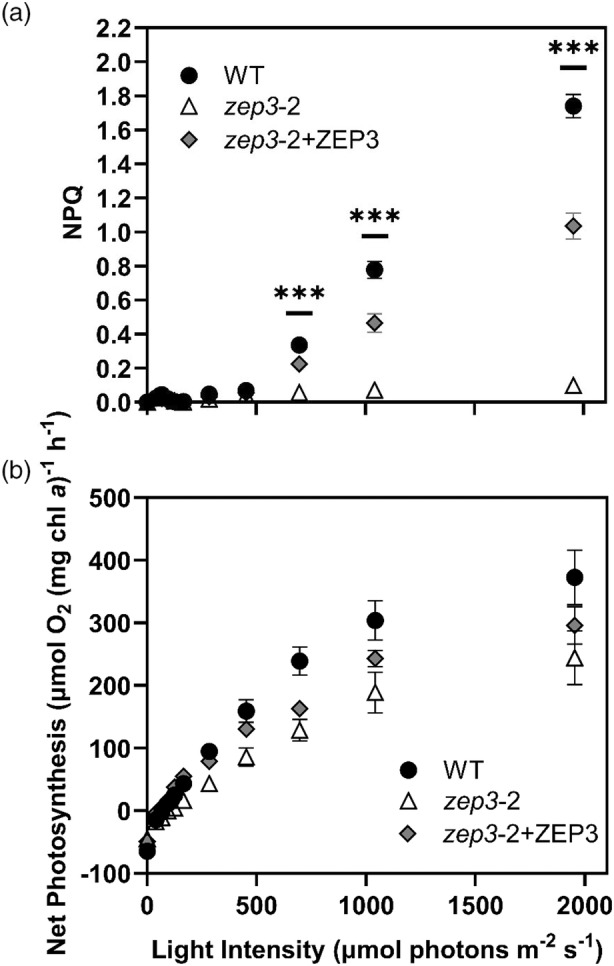
Non‐photochemical quenching (NPQ) capacity and net photosynthesis of *Phaeodactylum* cultures during an P‐I curve. (a) NPQ measured with a DUAL‐PAM fluorometer. (b) Net photosynthesis derived from gross oxygen evolution rates were measured with a FireSting oxygen probe, corrected for dark respiration rate, and normalized to total chlorophyll *a*. Cells were collected from sinusoidal light grown cultures and samples were taken within 2 h of solar noon. Dynamics were observed for *Phaeodactylum* WT, one *zep3* mutant strain, and one ZEP3 complemented strain. Points are averages with error bars representing one standard deviation (*n* = 3). Asterisks represent timepoints wherein all groups have statistically significant differences from one another, tested via an ordinary two‐way analysis of variance (anova) with a Tukey's multiple comparisons test (***P < 0.0005).

We sought to understand if reduced growth rates in variable light environments are due to a change in overall photosynthetic activity. Cells were sampled from sinusoidal grown cultures at ±1 h of solar noon. We chose solar noon for these assays as we sought to assign changes in physiology during excess light to changes in fitness. We performed simultaneous PAM fluorescence and oxygen evolution measurements to quantify changes in photosynthetic performance in a series of increasing light intensities. As expected from prior measurements, the NPQ capacity of *zep3* remains at a low level compared to WT, even at the highest light intensities (Figure 4a). The complemented strain showed an intermediate phenotype. While photosynthetic rates for the WT and complemented strains appeared higher than the KO strain at light intensities above 250 μmol photons m^−2^ sec^−1^ (Figure 4b), the calculated maximal rates of photosynthesis (*P*_max_) were statistically identical between all three strains (Table 1).

**Table 1:** Photosynthetic parameters derived from photosynthesis‐irradiance curves

Strain	*α* (μmol O^2^ mg chl. *a* ^−1^ h^−1^ (μmol photons m^−2^ sec^−1^)^−1^)	*I*_K_ (μmol photons m^−2^ sec^−1^)	*P*_max_ (μmol O^2^ mg chl. *a* ^−1^ h^−1^)
WT	0.61 ± 0.06^A^	442 ± 16^A^	389 ± 56^A^
*zep3‐2*	0.33 ± 0.04^B^	567 ± 68^B^	275 ± 60^A^
*zep3‐2* + ZEP	0.44 ± 0.04^A^	490 ± 76^A^	314 ± 31^A^

Strains were evaluated for the light limited slope of photosynthesis (*α*), the irradiance at saturation (*I*_K_) and maximal rates of photosynthesis (*P*_max_). Values are averages (*n* = 3) ± standard deviation. Letters indicate statistical differences between samples (*P* < 0.05).

Chl, chlorophyll.

Removal of *ZEP3* did affect photosynthetic rates in lower light fluxes. The calculated *α* parameter represents the efficiency of light harvesting during light‐limited photosynthesis. The *zep3‐2* mutant had a clearly reduced efficiency compared to the WT and complemented strains (Table 1), suggesting that the “locked” NPQ state reduced overall photosynthetic rate in LL. Correspondingly, *zep3* also had significantly higher irradiance for light saturation (*I*_K_). These results suggest the lower growth rates observed for the *zep3* mutant in variable light are due to its reduced capacities for light capture in sub‐saturating light.

### The capacity to change light harvesting efficiency in variable light is impaired in *zep3*

**Figure 5:**
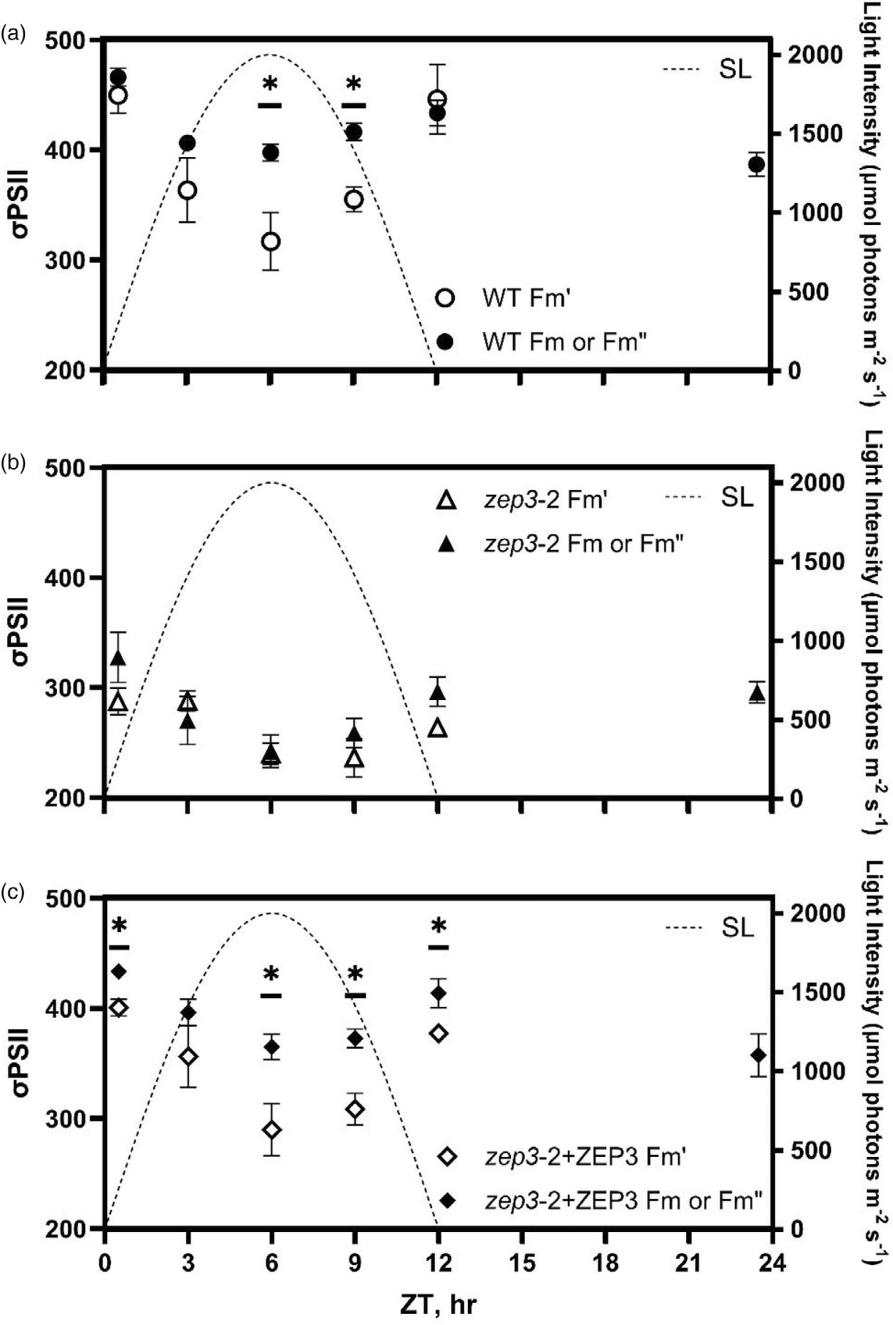
PSII functional absorption cross‐sections (σPSII) measured over a sinusoidal day. (a–c) Values of σPSII were measured immediately after sampling (white points, $F_{m}^{\prime }$) or following a 20 min low light relaxation (black points, *F*_m_ for predawn sample; $F_{m}^{\prime \prime }$ for samples collected during the day) with a Satlantic FIRe fluorometer. Measurements were performed for *Phaeodactylum* WT (a), one *zep3* mutant strain (b), and one ZEP3 complemented strain (c). The corresponding sinusoidal light condition is plotted using the right *Y*‐axis. Points are averages with error bars representing one standard deviation of biological replicates (*n* = 3). Asterisks represent statistically significant differences between the $F_{m}^{\prime }$ and *F*_m_ or $F_{m}^{\prime \prime }$ values, tested via unpaired *t*‐tests at each timepoint with Holm‐Šídák's multiple comparison method (*P < 0.05).

It was also of interest to us to investigate how the inability to rapidly regulate NPQ in variable light would affect the light harvesting capacity of *zep3* compared to the WT. We used Fluorescence Induction and Relaxation (FIRe) fluorometry to measure the PSII absorption cross‐section of cells sampled from cultures over the course of a sinusoidal day and night. This was done both immediately upon sampling, to approximate the *in situ* absorption cross‐section, and following a 20 min dark relaxation. The absorption cross‐section of WT a half hour after dawn was high and had similar values between *in situ* and dark‐relaxed states, at 450 and 466 Å^2^ quantum^−1^, respectively (Figure 5a). Toward solar noon, both *in situ* and dark‐relaxed absorption cross‐section values decreased relative to the values following dawn and rose again toward dusk. The *in situ* values changed from 450 Å^2^ quantum^−1^ after dawn to 317 at noon and back to 446 before dusk. Dark relaxation considerably recovered the absorption cross‐section value of the WT relative to the values taken immediately after sampling during the day. At noon, the average absorption cross‐section value taken upon sampling was 317 Å^2^ quantum^−1^, whereas the value after dark relaxation was 398. The absorption cross‐section decreased somewhat from half an hour before dusk to a half hour before the following dawn, from 446 to 387 Å^2^ quantum^−1^. These trends for WT were mimicked by the *zep3‐2* + ZEP3 complemented strain, albeit with slightly lower values across the day (Figure 5c).

In contrast to WT, the absorption cross‐section values of *zep3* had less of a dynamic range both over the course of the day and between dark‐relaxed and *in situ* states. Values for *zep3* were less than WT across the whole day; for instance, *zep3* showed a dark‐relaxed absorption cross‐section of 328 Å^2^ quantum^−1^ a half hour after dawn, compared to 466 for WT at that time (Figure 5b). The *in situ* value at noon for *zep3* was 242 Å^2^ quantum^−1^, and this compared to 287 after dawn is a much lower change than WT exhibited between these time points, which had a difference of 133 Å^2^ quantum^−1^. The dark‐relaxed values from the WT cultures in the middle of the day were much higher than the *in situ* values. This contrasts to the *zep3* cultures where the *in situ* and dark‐adapted values were fairly similar. This implies that *zep3* cells are impaired in adjusting their light harvesting capacity in response to natural light variation. This is further supported by the relative lack of change in the absorption cross‐section from just before dusk to the end of night, which remained at an average 296 Å^2^ quantum^−1^, in contrast to the larger decrease observed in WT over the night.

HPLC analyses of sinusoidal grown cultures also captured the ratio of chlorophylls for each strain. The ratio of chlorophyll *a*:*c* (or other accessory chlorophylls in other algal systems) can be used to estimate the relative abundance of antenna proteins compared to the photosystems (Cao et al., 2023). This is because chlorophyll *c* is found only in the antenna FCP proteins, whereas chlorophyll *a* is found in the antenna and photosystems. Samples were collected and analyzed as described above for DES. The WT cells had a chlorophyll *a*:*c* ratio of 6.3 (mol:mol), while the two *zep3* mutants and the complemented strain had ratios ranging from ~7.4 to 7.8 (Data S1). This suggests that the reduction in absorption cross section can be explained, in part, by a reduction in the total number of antennae serving photosystem II in the mutants versus the WT. However, similar values found between the KO mutants and complemented strains suggest that a change in antenna stoichiometry cannot completely explain the changes in antenna size (nor NPQ capacity). We note that the changes in stoichiometry do not explain the changes in reversible NPQ described above.

### Inducible ZEP3 expression causes dose‐ and time‐dependent NPQ capacity

**Figure 6:**
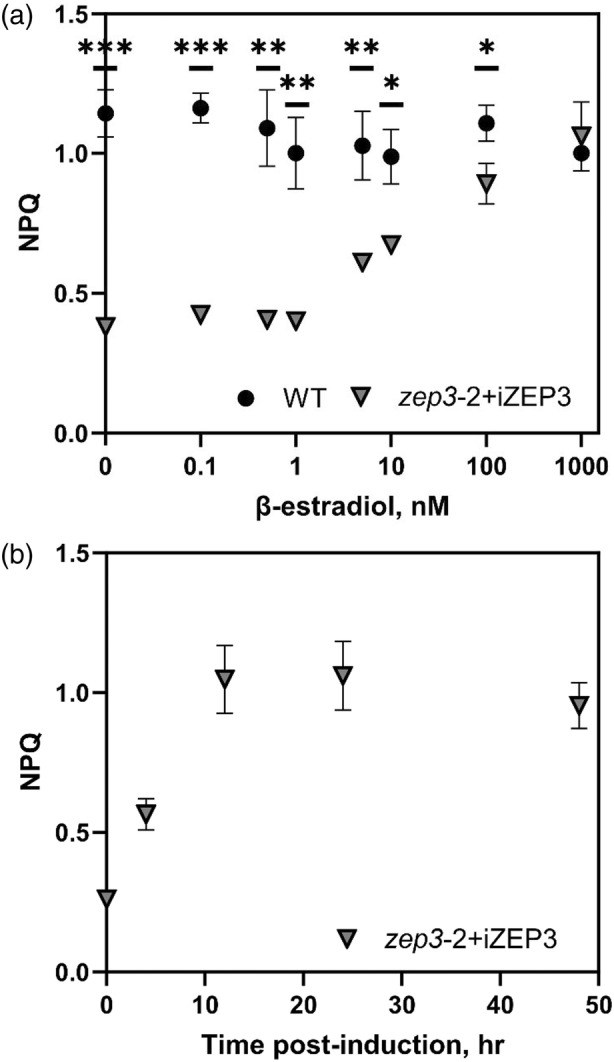
Dose‐ and time‐dependent tuning of non‐photochemical quenching (NPQ) capacity via chemical induction of *ZEP3* expression. An inducible ZEP3 *Phaeodactylum* strain (*zep3*‐2 + iZEP3) was created via complementation of the *zep3*‐2 mutant with a construct containing the native ZEP3 gene under the control of a β‐estradiol‐inducible synthetic promoter. (a) The NPQ of the iZEP3 strain and WT was measured with an IMAGING‐PAM at different β‐estradiol concentrations after 24 h. (b) The NPQ of the iZEP3 strain was measured over 48 h at 1000 nm β‐estradiol. Cells were incubated and assayed in 24 well plates with 2 ml of f/2 medium to which was added 2 μl of β‐estradiol at the needed concentration or 2 μl of ethanol as a vehicle control. Points are averages with error bars representing one standard deviation of biological replicates (*n* = 3). Asterisks represent statistically significant differences between WT and the iZEP3 strain, tested via unpaired *t*‐tests at each timepoint with Holm‐Šídák's multiple comparison method (*P < 0.05, **P < 0.005, ***P < 0.0005).

We then wanted to investigate if we could tune the level of reversible NPQ by adjusting levels of ZEP3. To do this, we modulated the expression of *PtZEP3* using the inducible expression system previously developed in our lab for *Phaeodactylum* (Kassaw et al., 2022). This previous work showed target protein accumulation correlated with the addition of increasing amounts of a chemical inducer (β‐estradiol). To that end, we complemented the *zep3‐2* mutant by putting the native coding sequence of *ZEP3* under the control of an inducible promoter driven by a synthetic β‐estradiol‐responsive transcription factor (Figure S5a). We obtained several inducible complemented strains confirmed by PCR amplification of the gene sequence as well as the inducible promoter sequence (Figure S5b). The NPQ capacity of these strains was tested with an IMAGING‐PAM over 48 h and over a β‐estradiol inducer range of 0–1 μm. An inducible *ZEP3* (iZEP3) strain subjected to this inducer range for 24 h showed a dynamic variation of NPQ capacity of 0.6 to 1.06 from 5 nm to 1 μm β‐estradiol (Figure 6a). The lowest inducer concentrations of 0.1 to 1 nm did not result in a detectable change in NPQ relative to the vehicle control of ethanol (NPQ ~0.38). In comparison, wild type displayed relatively consistent NPQ capacity in the tested range of β‐estradiol (range = 0.99–1.14). This is comparable to that of the iZEP3 strain at the highest used β‐estradiol concentration (Figure 6a). Induction of NPQ in the iZEP3 strain was also time‐dependent as high NPQ capacity at the highest β‐estradiol concentration was observed 12 h after induction and remained high through 48 h (Figure 6b). Notably, some increase in NPQ capacity was already observed after 4 h of induction (Figure 6b). The broader dynamic range of NPQ capacity in the iZEP3 strain can be seen across both time and dosage, reiterating that high NPQ is reached at 12 h and dynamic range is observed from 5 nm to 1 μm β‐estradiol (Figure S6).

## DISCUSSION

NPQ is a collective term for the quenching of chlorophyll fluorescence. This is achieved via an array of molecular mechanisms to dissipate absorbed photons upstream of the photosystem reaction centers. These mechanisms have been termed: energy‐dependent (qE, Wraight & Crofts, 1970), LCNP‐SOQ‐ROQH1‐dependent (qH, Malnoë et al., 2018), photoinhibitory (qI, Krause, 1988), chloroplast avoidance movement (qM, Dall'Osto et al., 2014), state transition (qT, Bonaventura & Myers, 1969) and zeaxanthin‐dependent quenching (qZ, Nilkens et al., 2010). Other factors, such as electrochemical gradients (Cardol et al., 2010), ion homeostasis (Kunz et al., 2014), and UV (Allorent et al., 2016) contribute to NPQ capacities and kinetics. Here we will focus on the two major mechanisms, qE and qZ, as these have also been identified to play significant photoprotective roles in algae and particularly in diatoms.

The well‐characterized qE response in plants is driven by an accumulation of protons in the thylakoid lumen. The acidification results in changes in the supercomplex structure of PSII, which favors energy transfer to lutein in LHCII and the dissipation of energy as heat (Ruban et al., 2007). Luminal acidification causes the LHC family protein PsbS to bind to major and minor antennae complexes to accelerate this process (Sacharz et al., 2017). Green algae have been shown to possess a similar cascade of events. Green algae (Peers et al., 2009), mosses (Alboresi et al., 2010) and some streptophytes (Gerotto & Morosinotto, 2013) possess LHCSR as well as PsbS. The more closely related the green alga is to a streptophyte, the more significant a role PsbS plays in NPQ, and conversely LHCSR plays a reduced role.

qZ is an NPQ component directly proportional to the “light‐dissipative” carotenoid present in the light harvesting antennae. The induction of qZ is significantly affected by qE, but its reversal is ∆pH independent. In higher plants and green algae, this carotenoid is zeaxanthin. The VAZ cycle is described in the introduction of this manuscript.

Diatoms, hacrobians and alveolate algae possess LHCX and LHCX‐like proteins which are analogous to LHCSR. LHCX/LHCX‐like proteins accumulate in stress conditions like LHCSR and PsbS (Bailleul et al., 2010), but the mode of action which increases thermal energy dissipation is not yet clear (Giovagnetti & Ruban, 2017). The VAZ cycle can also be observed in diatoms (Lohr & Wilhelm, 1999), but diatoms, haptophytes and dinophytes also utilize an independent one‐step xanthophyll cycle. The Ddx:Dtx cycle has also been suggested as solely responsible for NPQ in diatoms (Blommaert et al., 2021). A unique feature of this cycle, presumably due to chlororespiration maintaining low pH, is a residual pool of diatoxanthin that exists in darkness. Here we aimed to identify a gene responsible for the Ddx/Dtx cycling in diatoms and investigate its importance in NPQ dynamics.

### ZEP3 is required for the epoxidation of diatoxanthin and NPQ relaxation

We focused our physiological characterization on two *zep3* knockout (KO) strains along with a single strain that had been complemented with the native *ZEP3* gene. Both the WT and *ZEP3* complemented strains showed rapidly inducible and reversible NPQ when challenged with excess light (Figure 1). The mutant strains had considerably lower inducible NPQ that did not revert following a shift to LL. Correspondingly, the WT and the complemented strain showed the expected change of the xanthophyll cycle pigments—an increase in Dtx content in excess light and its reversal to Ddx in LL. The *zep3* mutants started the excess light treatment with elevated Dtx, followed by additional conversion of Ddx to Dtx during excess light and very little re‐epoxidation during LL recovery (Figures 1 and 2; Figure S3). This result led us to assume that ZEP3 is likely the Dtx epoxidase enzyme responsible for reversing NPQ back to the light harvesting state. This illuminates the diversification of function for the ZEP family in Ochrophytes as ZEP1 is known as an integral part of the fucoxanthin biosynthesis pathway (Bai et al., 2022). We highlight that we did not measure the catalytic activity of the *ZEP3* gene product which will be required to irrevocably assign this protein as the Dtx epoxidase. The role of ZEP2 was not investigated in our work. We note here that others have recently also proposed that ZEP3 is the Dtx epoxidase in diatoms (Græsholt et al., 2024), with their KO mutants also showing similar patterns of NPQ and Dtx:Ddx cycle to what we have reported here.

### ZEP3 is required for maximum growth rates in variable light regimes

Traditionally, static light regimes with 24 h day or square wave 12 h day:night cycles, have been used in laboratories to study photosynthesis. Increasingly, intermittent, variable or more natural light regimes are being used to study the contribution of individual proteins and pathways involved in excess energy dissipation pathways such as alternative electron transport (Allahverdiyeva et al., 2013; Andersson et al., 2019; Nawrocki et al., 2019). We initially characterized the growth of the *zep3*, complemented, and WT strains in constant light. LL and HL culturing conditions revealed no changes in relative growth rates between the three groups (Figure S4). We hypothesized that very little NPQ is induced in these conditions due to physiological acclimation to the uniform light flux (Jallet et al., 2016).

However, NPQ is expected to play an important role in conditions where light fluxes exceed the ability for cells to utilize absorbed light energy for photosynthesis (Murchie & Ruban, 2020). We designed three different conditions to assay the effect of variable light on fitness (growth rate). The first condition was a SL condition, mimicking conditions in the upper water column on a sunny day with no mixing (Ware et al., 2020). The second condition used an IL series where cells move in and out of saturating light in a rapid fluctuation. The IL light series has been shown to induce massive NPQ capacity in diatoms (Ruban et al., 2004). The third condition is an estimate of mixing in an estuarine environment (EL) where cells quickly mix between the surface and depth. We used light attenuation and water column mixing rate data to recreate a light environment that could occur in an estuary. We found that each of these conditions resulted in a lower growth rate for the *zep3* mutants compared to the WT (Figure 3). The complemented strain recovered to WT growth rates in SL and IL light conditions. But we note the complemented strain was not statistically different from either WT or *zep3* mutants in the EL condition, though a similar trend to the other conditions was apparent. These results offer two major takeaways. Firstly, ZEP3 and the conversion of Dtx back to Ddx is critical for maximum growth rates in dynamically changing light environments, such as those found in natural environments. Secondly, simulating natural light environments can elucidate key processes that are required for maximal growth rates of algae. While there are clear demonstrations that algae with the inability to induce NPQ have reduced fitness in excess light or rapidly changing light due to increased oxidative damage (Bailleul et al., 2010; Peers et al., 2009), our results demonstrate the capacity to reverse NPQ is also required for maximal growth in variable light.

### ZEP3 is essential for high rates of photosynthesis

To test whether the decreases observed in cellular fitness in variable light were the result of impaired photosynthetic performance, we assayed photosynthesis via simultaneous PAM fluorescence and oxygen evolution measurements. Photosynthesis‐irradiance curves have long been used to estimate the efficiency of light harvesting (MacIntyre et al., 2002). We found WT NPQ was induced at light fluxes above 500 μmol photons m^−2^ sec^−1^ (Figure 4). As expected, the *zep3‐2* mutant showed little change in NPQ capacity throughout the experiment while the complemented strain showed an intermediate phenotype. The *zep3‐2* mutant showed reduced levels of oxygen evolution compared to the WT as indicated in Table 1. Therefore, ZEP3 is required to maximize the efficiency of light harvesting and being locked in an NPQ state (high Dtx) results in lower rates of photosynthesis. This is supported by our calculations of NPQ_(t)_ that show the overall capacity of NPQ is similar between the knockouts and WT, even though our assays begin with the mutants in a more quenched state (Tietz et al., 2017). Similarly, the *npq2* mutant in Arabidopsis, which is devoid of the zeaxanthin epoxidase, has lower quantum yields of PSII (ΦPSII) at LL intensities but not at HL, compared to WT (Ware et al., 2016). Our work demonstrates that *zep3* mutants are impaired in recovery of ΦPSII following a HL challenge (Figure S3d), which provides further justification for the lower cellular fitness (growth rate) we observed in variable light.

### Natural light regimes require dynamic changes in the cross section of PSII

It is assumed that NPQ processes exert a strong influence on the variable fluorescence observed in natural phytoplankton communities, with clear reductions of fluorescence yields associated with excess light (Gorbunov & Falkowski, 2022). Our estimations of photosynthetic performance for cells grown in SL suggested that *zep3* had reduced light harvesting efficiencies (Figure 4), which was likely influenced by cells being locked in an induced NPQ state. NPQ in *Phaeodactylum* has been correlated with a decrease in the functional absorption cross section of PSII (σPSII, Buck et al., 2019; Giovagnetti & Ruban, 2017). The σPSII has also been demonstrated to vary significantly during the course of SL cultivation, presumably due to the induction/reversal of NPQ (Jallet et al., 2016). We probed whether the variations in the σPSII were influenced by ZEP3. Cultures were assayed within 30 sec of removal from *in situ* SL conditions to estimate the σPSII *in vivo*. Estimations of the σPSII suggested that ZEP3 is required *in situ* to alter the σPSII (Figure 4). WT and ZEP3 complemented cells had a σPSII almost twice as large as ZEP3 cells at dawn and dusk. At solar noon, in conditions of 2000 μmol photons m^−2^ sec^−1^, there was only a small difference in the σPSII of ZEP3 to the WT *in situ*. In lower light environments after solar noon, ZEP3 cells have fewer antenna proteins delivering excitation energy to PSII, which is supported by the reduced light harvesting efficiency inferred from oxygen evolution measurements (Figure 4). Reduced rates of oxygen evolution are therefore likely a contributing factor to the reduced fitness of ZEP3 KOs. One of the reasons for reduced photosynthetic performance is the irreversible accumulation of diatoxanthin (Figure 2a), which reduces the light harvesting capacity of *zep3* by irreversibly decreasing σPSII. Overall, this illustrates the importance of reversing from NPQ to a light‐harvesting state in a natural environment. Based on measurements of chlorophyll *a*:*c* ratios, *zep3* and complemented strains do have a lower FCP content compared to WT cells during growth in SL. Future studies should investigate the role of reversible NPQ on photoacclimation in diatoms, but we restrict our discussion here to the role of ZEP3 in regulating NPQ.

### Synthetic circuits can be utilized to control ZEP3 expression in a time and dose‐dependent manner

NPQ is a target for engineering photosynthetic efficiency (Murchie & Ruban, 2020). The concept associated with this engineering target is that dense algal cultures or high‐density canopies lead to highly variable light environments that change faster than native NPQ mechanisms can turn on and off. Therefore, fast transitions from excess light to LL may result in lower light harvesting capacity due to the persistence of NPQ. Indeed, work in cyanobacteria and plants has shown that the deletion of NPQ capacity (Peers, 2015) or tuning of NPQ induction and relaxation can lead to increased biomass accumulation in agricultural situations or dense algal cultures (De Souza et al., 2022; Kromdijk et al., 2016; Perin et al., 2023). We investigated if the level of NPQ could be tuned in *Phaeodactylum* using a synthetic promoter system. We modified our inducible expression system (Kassaw et al., 2022) to incorporate the native ZEP3 coding sequence under the control of a β‐estradiol‐dependent promoter into *zep3* cells (*zep3*‐iZEP3; Figure S5). Within 4 h of induction, the phenotype of reversible NPQ could be observed in the 100–1000 nm β‐estradiol treatments. Additionally, β‐estradiol concentrations as low as 5 nm partially restored NPQ reversal within 12 h. This shows NPQ can be controlled like a rheostat in a time and dose dependent manner. Using synthetic circuits to control the expression of photosynthetic and photoprotective enzymes could allow algal cultivators to adjust the photosynthetic performance of algae in near real‐time depending on the growth phase of cultivated algae. Future studies could investigate if biomass or bioproduct formation could be enhanced in mass cultures of algae using synthetic control of NPQ capacity.

## CONCLUSION

Diatoms contend with mixing in the water column and resulting fluctuations in light levels, which provides environmental selection for efficient NPQ. In several ways, diatoms have less complicated mechanisms of NPQ than land plant and green algal counterparts. Diatoms do not exhibit state transitions (qT) and therefore rely heavily on xanthophyll cycling (qE) for NPQ on shorter time scales. While in plants, xanthophyll cycling involves the generation of two de‐epoxidated xanthophylls, antheraxanthin and zeaxanthin, diatoms only remove one epoxide ring from Ddx to form Dtx for photoprotection. Our work demonstrates that a member of the *Phaeodactylum* ZEP family, ZEP3, likely functions as the diatoxanthin epoxidase involved in NPQ. This enzyme is needed for rapid reversal of NPQ, cellular fitness in variable light environments, and normal photosynthetic rates and photo‐physiological parameters under HL. We also demonstrate that inducible expression of ZEP3 leads to variable levels of NPQ. This has potential ramifications for engineering photosynthetic efficiency in industrial culture of diatoms in different light regimes of interest.

## MATERIALS AND METHODS

### Growth conditions

*Phaeodactylum tricornutum* CCAP 1055/1 was grown axenically in Instant Ocean artificial seawater medium (35.95‰ salinity), supplemented with 0.03% Proline A and Proline B micronutrient stocks. Cultures were maintained in exponential growth phase (approx. 5 × 10^4^–2 × 10^6^ cells ml^−1^) throughout this study. Cultures were grown under constant shaking (VWR shaker model 3500, shaker setting 4), 18°C, and ambient air. Light intensities were measured using the Walz ULM‐500 universal light meter. Five different light regimes were used to assay fitness in this study. Two are 24 h constant light regimes at HL (450 μmol photons m^−2^ sec^−1^) or LL (60 μmol photons m^−2^ sec^−1^). The remaining three are 12 h D:N cycles with variable light intensity. The SL regime follows this formula:

$$\text{Incident Light Flux}\left(t\right)=A_{\max}\times \sin\;\left(2\pi \times f\times t\right)$$

where *t* = *h* after dawn, *A*_max_ = 2000 μmol photons m^−2^ sec^−1^, *f* = 1/(DayLength [h]) = 1/24. The light changes in intervals of 5 min. The IL regime followed a square wave pattern (day: 120 sec 1000 μmol photons m^−2^ sec^−1^, 120 sec darkness, etc.). The EL regime was based around environmental conditions measured in two different estuaries. Water turbidity measurements in the Chesapeake Bay suggested less than 5% of incident light is available below 1 m depth (Carter & Rybicki, 1990). Measurements of water movement in the Columbia River estuary suggested an average mixing depth of 7 m and an average mixing efficiency of 0.36. Combining these two studies, we then simplified the complexity of mixing and assumed that cells would spend 1/7th of their time in the light, mixing up into full sunlight and then down again into darkness for the remaining 6/7th of the time. The EL was programmed as a series of short spikes (day: 1 sec intervals of 37, 99, 269, 2 s of 2000 μmol photons m^−2^ sec^−1^, followed by a mirrored descending pattern and then 22 s of darkness, etc.).

### ZEP3 identification

Published nucleotide sequences of *P. tricornutum* from the Ensembl protists genome browser was used for identifying target genes. The *Arabidopsis thaliana* Zeaxanthin epoxidase (ZEP) gene (TAIR locus AT5G67030) was used as the query. The ZEP3 gene is annotated as Phatr3_J10970 in the *P. tricornutum* published genome (ASM15095v2).

### Generating HDR mutants of ZEP3

CRISPR‐Cas9‐mediated homology directed repair (HDR) was used to generate successful knock‐outs of the *ZEP3* gene in *P. tricornutum* (Figure S1). Two independent guide RNA sequences (Data S2 for all sequences and primer details) were designed utilizing the motif [5′‐N20‐NGG‐3′] against the *P. tricornutum* genome. Oligo design, annealing and synthesis were performed as previously described (Bai et al., 2022) using BsaI‐HF®v2 (NEB) digested vector pKS diaCas9_sgRNA (Addgene #74923). The homology donor plasmid was constructed via Gibson assembly (ThermoFisher USA GeneArt; Gibson Assembly HiFi master Mix #A46628) by fusing 1 kb 5′ homologous arm, ble cassette and 1 kb 3′ homologous arm onto the BamHI (NEB) digested backbone of pUC57. Homology arm sequences and oligos sequences are detailed in Table S1. Linearization of the homology donor and pKS diaCas9_sgRNA plasmids was performed by KpnI‐HF® and NdeI (NEB, USA) restriction enzymes, respectively. For each electroporation, 100 ml of mid‐exponential growth phase cells were harvested at 4°C, 2500 ***g***, 10 min. The supernatant was carefully discarded prior to four washes of the pellet in ice cold filtered 0.375 m sorbitol. Cells were resuspended in a final 100 μl of ice‐cold 0.375 m sorbitol. Forty micrograms of heat denatured salmon sperm (1 min 100°C) and 2 μg of linearized homology donor and pKS diaCas9_sgRNA plasmids were added to cells and incubated on ice for 10 min. This mixture was transferred to a 2 mm ice cold electroporation cuvette (Bio‐Rad, Hercules, CA, USA). Transformation of WT *P. tricornutum* was performed via electroporation (Bio‐Rad GenePulser Xcell; exponential decay, 0.5 kV, 25 μF, 400 Ω). Cells were immediately mixed with 10 ml of 18°C f/2 medium in a 15 ml conical tube. Cells were placed under 30 μmol photons m^−2^ sec^−1^ for 24 h for transformation recovery. Cells were then spread onto selection plates (f/2 + 100 μg ml^−1^ zeocin 1% agar) in 24 h 80 μmol photons m^−2^ sec^−1^ light conditions.

### Complementation of knockout mutants with native ZEP3 gene

For complementation of knockouts, Phusion® HF DNA polymerase (NEB, USA) mediated PCR amplification of the ZEP3 gene included all exon, intron, promotor and terminator regions of the WT native ZEP3 (primers 4/21, Table S1). PCR amplification of a Blasticidin S deaminase (BSD) cassette was performed as previously described (Bai et al., 2022). Two micrograms of the BSD and ZEP3 amplicon sequences were used in electroporation, as described above. Selection was performed by plating transformants on f/2 + 10 μg ml^−1^ blasticidin S HCl (ThermoFisher Scientific, USA) 1% agar plates under 24 h 80 μmol photons m^−2^ sec^−1^ light conditions.

### β‐Estradiol inducible ZEP3 expression

First, overlapping PCR was used to generate a ZEP3‐NosT fragment. ZEP3 and NosT sequences were PCR amplified separately using primer pairs 9/10 and 11/12, respectively. Overlapping PCR products were generated using primers 9 and 12 flanked by AvrII and SbfI restriction sites (Table S1). Phusion® HF DNA polymerase (NEB) was used for all amplification steps. The ZEP3‐NosT PCR product was sub‐cloned into the pJET vector using the Blunt‐End Cloning Protocol (ThermoFisher K1232) and the PCR product of primers 13 and 14 (Table S1) was sequence verified. The ZEP3‐NosT fragment was then cloned into the TKK031 vector (Kassaw et al., 2022) by digesting the plasmid with AvrII, SbfI and BamHI. The pJET_ZEP3‐NosT plasmid was digested with AvrII and SbfI restriction enzymes (NEB). These were then ligated with T4 DNA ligase (NEB) according to manufacturer instructions. For double selection in our knockout strain background, we needed to change the antibiotic resistance gene. For this, we transferred the inducible ZEP3 expression cassettes from TKK031 to a PtPUC3 background containing a blasticidin resistance cassette (Kassaw et al., 2022). BstXI and SbfI unique restriction sites were used for this purpose. Transformation and screening of strains were performed as described above.

### Genotyping

Cell biomass (∼10^6^ cells) was heated at 85°C in 50 μl TE buffer (50 mm Tris HCl, 10 mm EDTA, pH 8.0) for 10 min. Samples were cooled on ice, before adding 50 μl of TE buffer and vortexed. Samples were centrifuged at 10 000 ***g*** at 18°C for 10 min. The supernatant was carefully removed and frozen at −20°C until required for PCR analysis. One microlitre of extract was used as a DNA template for 1 × Gotaq® Green Master Mix (Promega, USA) 25 μl volume PCRs. Full primer sequences listed in Table S1 were used for sequence verification. All gel electrophoresis experiments were performed in 1% agarose, 0.01% gel red, TAE buffer (40 mm Tris, 20 mm acetic acid, 1 mm EDTA).

### Cell density and growth rate measurements

A BD Accuri C6 flow cytometer gated for chlorophyll autofluorescence (excitation 488 nm, emission 670 nm) was used to measure culture cell densities. One millilitre of cells was passed through a 30 μm filter prior to sampling. Flow cytometric cell counting was performed at a flow rate of 36 μl min^−1^ for 40 sec, with a minimum number of 1000 cells measured for each sampling timepoint. Cultures grown under a 12 h D:N photoperiod were measured within ±1 h of solar noon each day. Cell densities were used to calculate the intrinsic growth rate (μ in units of day^−1^) using the methodology described by Cao et al. (2023).

### Chlorophyll extraction and quantification

Cells were collected and TWEEN 20 (0.01% final concentration, p7949; Sigma‐Aldrich) was added. Samples were harvested after centrifugated at 10 000 ***g*** for 10 min at 18°C and discarding the supernatant. Chlorophylls were extracted in 100% HPLC grade methanol for 10 min in the dark. Cell debris was removed via centrifugation at 20 000 ***g*** for 10 min at 18°C (Porra, 2002). Concentrations of chlorophyll *a* and *c* were calculated using established absorbance and extinction coefficients (Ritchie, 2008).

### High performance liquid chromatography

Cultures were grown under the SL regime (see section “Growth conditions”). At 1 h post dawn, cultures were collected and transferred to LL (75 μmol photons m^−2^ sec^−1^) for 60 min to relax NPQ and ensure maximal diatoxanthin epoxidation. Exponentially growing cultures (average 3.95 × 10^5^ cells ml^−1^) were sampled at a volume corresponding to a total 7.5 μg chlorophyll *a*. Cells were then exposed to 2000 μmol photons m^−2^ sec^−1^ for 15 min before harvesting the same volume. After placement at 70 μmol photons m^−2^ sec^−1^ for the 15 and the 30 min timepoints, cells were harvested again. Cells were collected via gentle vacuum onto a GF/6 filter. Cells were harvested in the current light condition before being placed in a cryovial and frozen in liquid nitrogen within 15 sec of sampling and then lyophilized. Pigments were extracted from the filters and analyzed by HPLC using HPLC system II as described in Bai et al. (2022).

### PAM NPQ state estimations corresponding to HPLC analysis

Cultures were grown under the SL regime. At 1 h post dawn, cultures were collected and transferred to LL (75 μmol photons m^−2^ sec^−1^) for 60 min to relax NPQ and ensure maximal diatoxanthin epoxidation. Exponentially growing cultures were sampled at a volume corresponding to a total 2.5 μg chlorophyll *a* and collected on artificial leaf disks (glass fiber prefilters) pre‐soaked in 18°C f/2 media. *F*_v_/*F*_m_ was measured (saturating pulse [SP] of 10 000 μmol photons m^−2^ sec^−1^, duration 0.6 sec). Cells were illuminated with 1994 μmol photons m^−2^ sec^−1^ for 15 min (SP applied every 150 sec). The actinic light level was reduced to 72 μmol photons m^−2^ sec^−1^ for 30 min with an SP applied every 150 sec. All PAM measurements were performed using a DUAL‐PAM system (Heinz Walz GmbH, Effeltrich, Germany).

### PAM NPQ state estimations for complemented strain screening

Cultures were grown under the HL regime. Cells were sampled at a volume corresponding to a total 2.5 μg chlorophyll *a* and then placed in 30 μmol photons m^−2^ sec^−1^ white light conditions for 20 min prior to screening. They were then collected on artificial leaf disks and subjected to the same light regime as described above (see “PAM NPQ state estimations corresponding to HPLC analysis” section).

### PAM NPQ state estimations for LL measurements

Cultures were also grown under the LL regime. Cells were sampled at a volume corresponding to a total 7.5 μg chlorophyll *a* and then collected on artificial leaf disks pre‐soaked in 18°C f/2 media. *F*_v_/*F*_m_ was measured (SP of 10 000 μmol photons m^−2^ sec^−1^, duration 0.6 sec). Cells were illuminated with 1996 μmol photons m^−2^ sec^−1^ for 10 min (SP applied every 60 sec). The actinic light level was reduced to 79 μmol photons m^−2^ sec^−1^ for 10 min with an SP applied every 120 sec. NPQ_(t)_ values were calculated according to the methodology reported in Tietz et al., 2017.

### Simultaneous PAM fluorometry and oxygen evolution measurements

The equipment set‐up for simultaneous measurements was the same as previously described (Jallet et al., 2016). All sampling and measurements were performed at 18°C. Samples collected ±2 h of solar noon. Chlorophyll quantification was performed as described above. Samples were collected via centrifugation (18°C, 2500 ***g***, 10 min, with a final concentration of 0.01% Tween 20). The supernatant was discarded and cells were resuspended in pre‐cooled, air saturated f/2 media. Two millilitre of cells at a concentration of 4 μg chl *a* ml^−1^ were used for measurements. The stopper was lowered to purge air from the sample, leaving a 1.4 ml volume for measurements. Cells were exposed to darkness for 5 min to measure dark respiration rates. Fifteen minutes of blue actinic light (79 μmol photons m^−2^ sec^−1^, BL int 5) was used to relax NPQ. Immediately following the relaxation, the actinic light was turned off and *F*_v_/*F*_m_ was measured (SP of 10 000 μmol photons m^−2^ sec^−1^, duration 0.6 sec). Cells were then exposed to 2 min steps of increasing actinic light intensities of 40, 67, 95, 125, 166, 284, 453, 697, 1042 and 1953 μmol photons m^−2^ sec^−1^, with an SP applied at the end of each step. SPs were delivered to derive *F*_o_, *F*, and *F*_m_, with these parameters used to calculate NPQ and ΦPSII according to published equations (Maxwell & Johnson, 2000). Gross oxygen evolution capacities were normalized to chl *a* concentrations after offsetting the dark respiration rates. The maximal rate of photosynthesis (*P*_max_), minimum light at saturation (*I*_K_) and the quantum efficiency of photosynthesis (*α*) were calculated as described in Ware et al., 2020.

### *In situ* estimations of functional absorption cross sections of PSII

Exponentially growing cultures were re‐established at 50 000 cells ml^−1^ at solar noon the day preceding measurements. At the specified timepoints, 1 ml of culture was transferred to a 1 cm quartz cuvette. The functional absorption cross section of PSII (σPSII; Å^2^ quantum^−1^) was measured within 5 sec of sampling using a Satlantic FIRe Fluorometer (excitation at 450 nm, emission at 678 nm, Satlantic LP, Halifax, Canada). A 1.1 ml sample was collected from the same culture and placed under 30 μmol photons m^−2^ sec^−1^ white light for 20 min. *σ*_PSII_ from a 1 ml subsample of this was measured after the LL relaxation to estimate the $F_{m}^{\prime \prime }$ state. Fireworx software (https://sourceforge.net/projects/fireworx/) was used to calculate *σ*_PSII_ from the raw fluorescence data. Sampling timepoints correspond to 0.5, 3, 6, 9, 11.5 and 23.5 h after dawn during a 12 h day:night SL regime (2000 μmol photons m^−2^ sec^−1^ maximum).

### Inducible ZEP3 expression NPQ relaxation dynamics

Cultures were maintained under 450 μmol photons m^−2^ sec^−1^, 18°C, 120 rpm, atmospheric air. For screening of NPQ induction and recovery phenotypes, cells were diluted to 100 000 cells ml^−1^ in a 24‐well plate in fresh f/2 supplemented with 5 mm sodium bicarbonate (from a 1 m stock) to a final volume of 2 ml. F/2 was accompanied with 2 μl of either 100% ethanol (control) or 0.1, 0.5, 1, 5, 10, 100 or 1000 nm final concentration β‐estradiol (dissolved in 100% ethanol) serially diluted from a 10 mm stock. 24‐well plates were placed in the same conditions. Cells were placed in 30 μmol photons m^−2^ sec^−1^ white light conditions for 20 min prior to screening. An imaging PAM (MAXI head, Heinz Walz Gmbh) was used to test for NPQ induction and relaxation properties. *F*_m_ and $F_{m}^{\prime }$ were derived from an SP (840 msec, 3600 μmol photons m^−2^ sec^−1^). The script used entailed an *F*_v_/*F*_m_ measurement, 5 min of 1250 μmol photons m^−2^ sec^−1^ (SP every 30 sec) and 15 min of 62 μmol photons m^−2^ sec^−1^ (SP every 60 sec for 3 min, subsequently every 120 sec for 12 min).

### Statistics

Experiments were performed in biological replicates (*n* = 3–6, see individual figure legends), except for Figure S1a. A repeated‐measure one‐way analysis of variance with a Tukey's HSD was performed to test for significant variations between group means for most experiments, except for Figures 5 and 6, which used unpaired *t*‐tests at each timepoint with Holm‐Šídák's multiple comparison method. Data represent mean values ± the standard deviation. Graphs and statistical analyses were performed in Prism version 10.1.2.

## AUTHOR CONTRIBUTIONS

GP, MAW and YB conceived the project and designed experiments. MAW, YB, AJP, TK and ML collected and analyzed data. GP and MAW wrote the original draft of the manuscript with contributions from AJP and ML. All authors contributed to review and editing of the manuscript and approved the final manuscript.

## CONFLICT OF INTEREST

The authors declare they have no competing interests.

## Supporting information


**Figure S1.** NPQ and genotype screening for multiple ZEP3 complemented lines.
**Figure S2.** Representative HPLC chromatograms from *Phaeodactylum* cultures during an NPQ induction experiment.
**Figure S3.** Chlorophyll fluorescence and photo‐physiological parameters of *Phaeodactylum* cultures during an NPQ induction experiment.
**Figure S4.** Maximal specific growth rates of *Phaeodactylum* cultures during different constant light regimes.
**Figure S5.** *Phaeodactylum* inducible ZEP3 strain design and genotype screening.
**Figure S6.** Tuning of NPQ capacity via chemical induction of ZEP3 expression.

**Data S1.** HPLC pigment analysis data.

**Data S2.** Gene sequences and primers excel file.
