# Supplementary material for: Identifying the gene responsible for non‐photochemical quenching reversal in Phaeodactylum tricornutum
Source: Plant J. 2024 Oct 30;120(5):2113–26. doi: 10.1111/tpj.17104 (PMC11629738; doi:10.1111/tpj.17104)
Supplement: Supplementary file 1 — Figure S1. NPQ and genotype screening for multiple ZEP3 complemented lines. Figure S2. Representative HPLC chromatograms from Phaeodactylum cultures during an NPQ induction experiment. Figure S3. Chlorophyll fluorescence and photo‐physiological parameters of Phaeodactylum cultures during an NPQ induction experiment. Figure S4. Maximal specific growth rates of Phaeodactylum cultures during different constant light regimes. Figure S5. Phaeodactylum inducible ZEP3 strain design and genotype screening. Figure S6. Tuning of NPQ capacity via chemical induction of ZEP3 expression. [file TPJ-120-2113-s003.docx]

**Supplementary Information for:**

**Identifying the gene responsible for NPQ reversal in *Phaeodactylum tricornutum***

Maxwell A. Ware, Andrew J. Paton, Yu Bai, Tessema Kassaw, Martin Lohr, Graham Peers


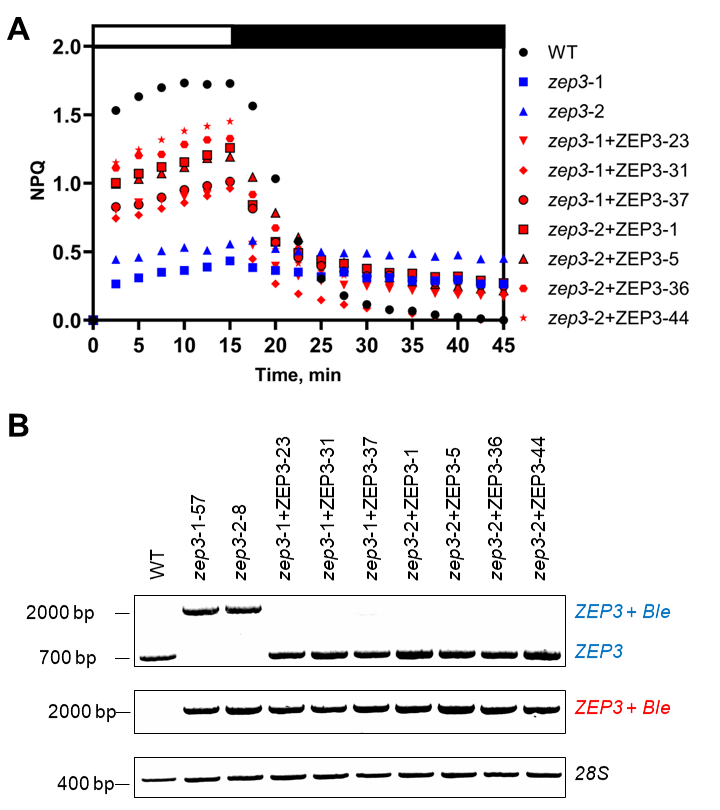


**Figure S1**

NPQ and genotype screening for multiple ZEP3 complemented lines. Data were taken for *Phaeodactylum* WT (black points), two *zep3* mutant strains (blue points), and seven ZEP3 complemented strains (red points). A, NPQ dynamics were measured with a DUAL-PAM fluorometer. Cells were exposed to 15 minutes of high light (2000 µmol photons m^-2^ s^-1^, white bar) and 30 minutes of low light (75 µmol photons m^-2^ s^-1^, black bar). 2.5 µg chlorophyll *a* from cells grown in high light were collected. Points represent values from single strains. B, Gel electrophoresis results showing expected band pattern for the seven ZEP3 complemented strains. Strain *zep3*-2+ZEP3-5 was selected as the complemented strain used in the main text. See Figure S1 for full explanation of expected band patterns.


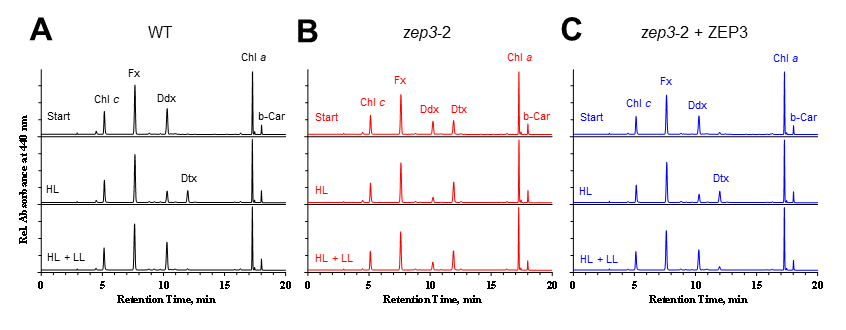


**Figure S2**

Representative HPLC chromatograms from *Phaeodactylum* cultures during an NPQ induction experiment. A-C, Samples were collected and analyzed for WT (A), one *zep3* mutant strain (B), and one ZEP3-complemented strain (C). Cells were exposed to 15 minutes of high light (HL, 2000 µmol photons m^-2^ s^-1^) and 30 minutes of low light (LL, 75 µmol photons m^-2^ s^-1^). HPLC results are shown from 0 (Start), 15 (HL), and 30 (HL + LL) minute sampling times. Pigment concentrations were assessed from relative absorption at 440 nm and normalized to chlorophyll *a*, as culture volumes corresponding to 2.5 µg of chlorophyll *a* at the “Start” sampling time were filter-collected at each time point. Pigment abbreviations: b-Car= β-carotene; Chl = Chlorophyll; Ddx = diadinoxanthin; Dtx = diatoxanthin; Fx = fucoxanthin.


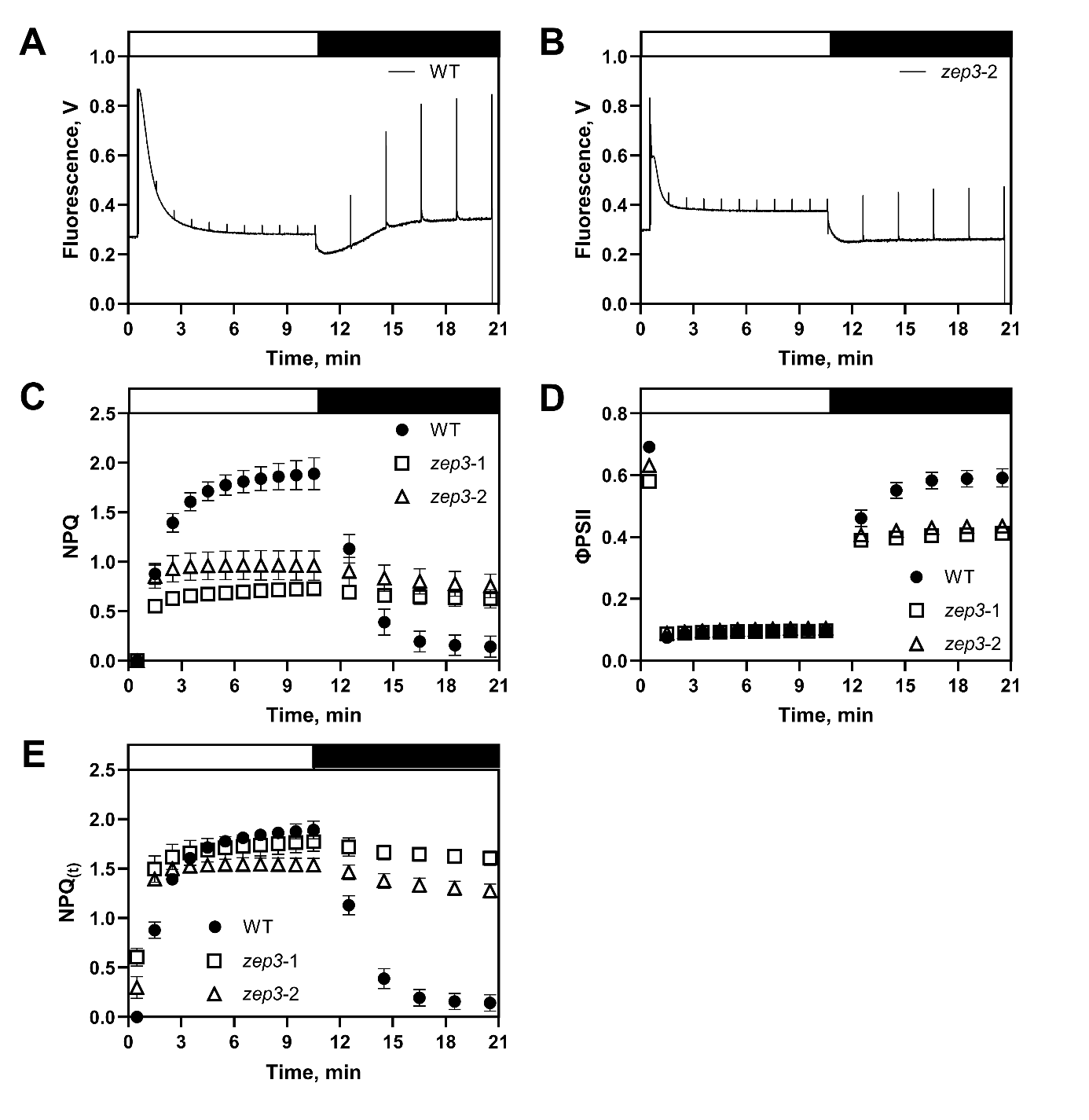


**Figure S3**

Chlorophyll fluorescence and photo-physiological parameters of *Phaeodactylum* cultures during an NPQ induction experiment. A and B, Chlorophyll fluorescence traces of WT (A) and a *zep3* mutant (B) measured with a DUAL-PAM fluorometer. C, D and E, NPQ (C), the quantum yield of PSII (D) and NPQ_(t)_ (E) of WT and two *zep3* mutants. Cells were exposed to 10 minutes of high light (2000 µmol photons m^-2^ s^-1^, white bar) and 10 minutes of low light (75 µmol photons m^-2^ s^-1^, black bar). Cells were sampled from low light-grown cultures at a volume corresponding to 7.5 µg chlorophyll *a*. Points are averages with error bars representing one standard deviation (n=3).


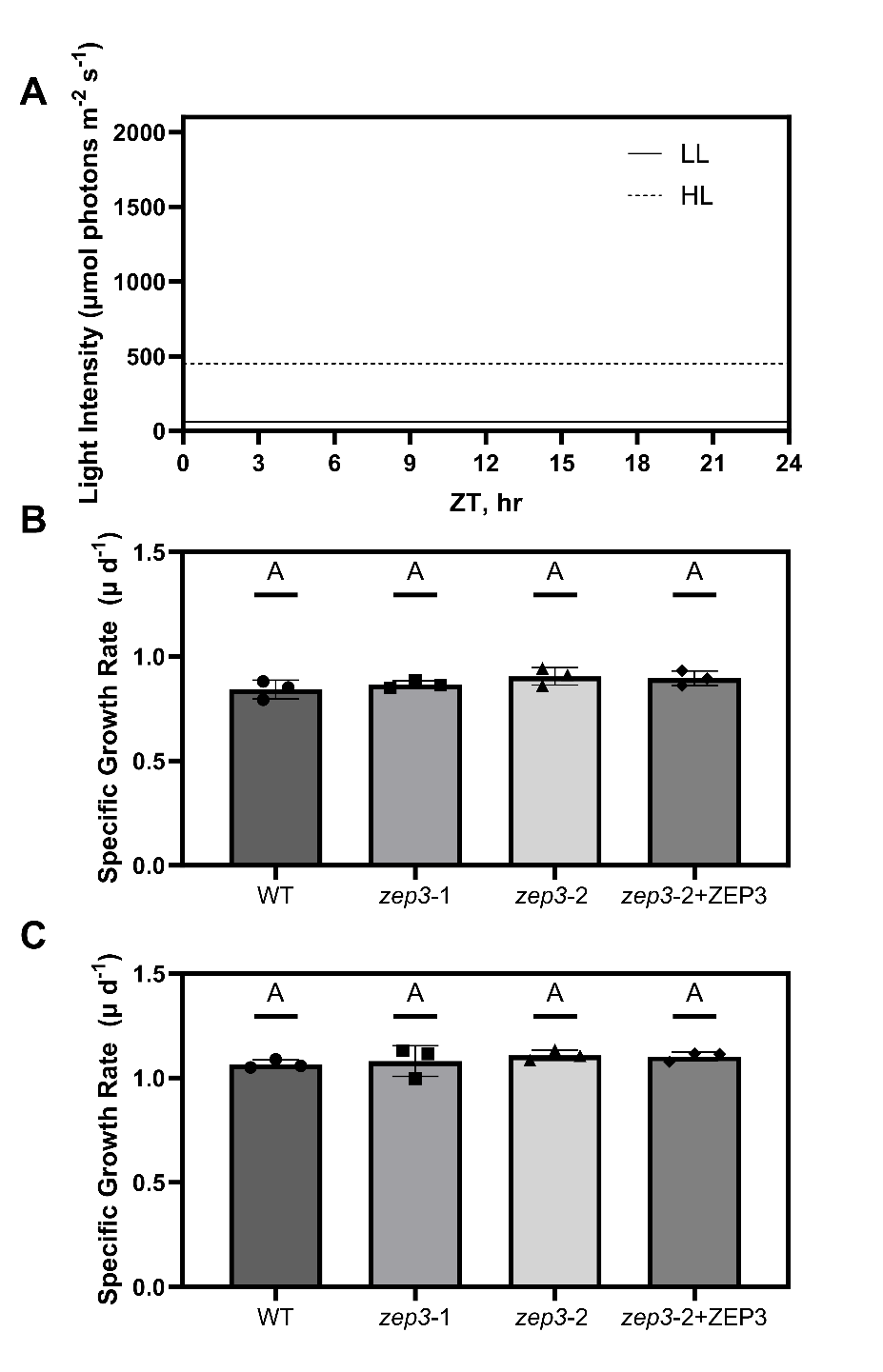


**Figure S4**

Maximal specific growth rates of *Phaeodactylum* cultures during different constant light regimes. A, Schematic of light regimes utilized in this experiment on a 24 hour time scale, namely low light (LL, 60 μmol photons m^−2^ s^−1^) and high light (HL, 450 μmol photons m^−2^ s^−1^). B and C, Specific growth rates of cultures grown in LL (B) and HL (C) regimes. Growth rates were observed for *Phaeodactylum* WT, two *zep3* mutant strains, and one ZEP3 complemented strain. Bars are averages with points from individual replicate cultures shown and error bars representing one standard deviation (n=3). Letters represent statistically different groups tested via a repeated measures one-way analysis of variance (RM-ANOVA) with a Tukey’s HSD.


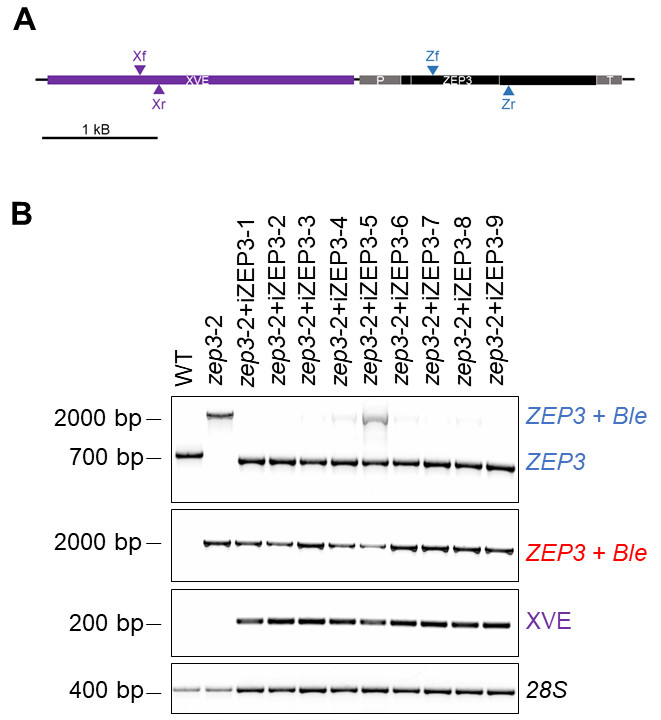


**Figure S5**

*Phaeodactylum* inducible ZEP3 strain design and genotype screening. A, Schematic of β-estradiol-inducible transcription factor gene construct (XVE) driving a complemented *Pt*ZEP3 cDNA sequence. Primer binding sites are indicated by arrowheads, with forward primers above the genes and reverse primers below the genes. There is a primer pair for ZEP3 amplification (Zf, Zr) and a primer pair for XVE amplification (Xf, Xr). “P” and “T” indicate the synthetic XVE-responsive promoter and a nosT terminator, respectively. A 1000 base pair scale bar is provided. B, Gel electrophoresis results for WT, the *zep3-2* mutant, and nine *zep3*-2+iZEP3 complemented strains. Labels on the right show expected band positions for different strains, with the colored labels corresponding to the primer colors in (A) and a 28S control amplification.. WT and iZEP3 complemented strains show a normal ZEP3 band, albeit at slightly different lengths corresponding to intron presence or absence, respectively, for the Zf/Zr amplification. Mutant and iZEP3 complemented strains show a ZEP3+*Ble* band for the BZf/BZr amplification while WT has no band. The iZEP3 complemented strains show an XVE band for the Xf/Xr amplification. Strain *zep3*-2+iZEP3-6 was selected as the complemented strain used in the main text. Relevant base pair migration positions from a Thermo Scientific 1 Kb Plus DNA ladder are given on the left. See Figure S1 for visual explanation of expected ZEP3+*Ble* band patterns.


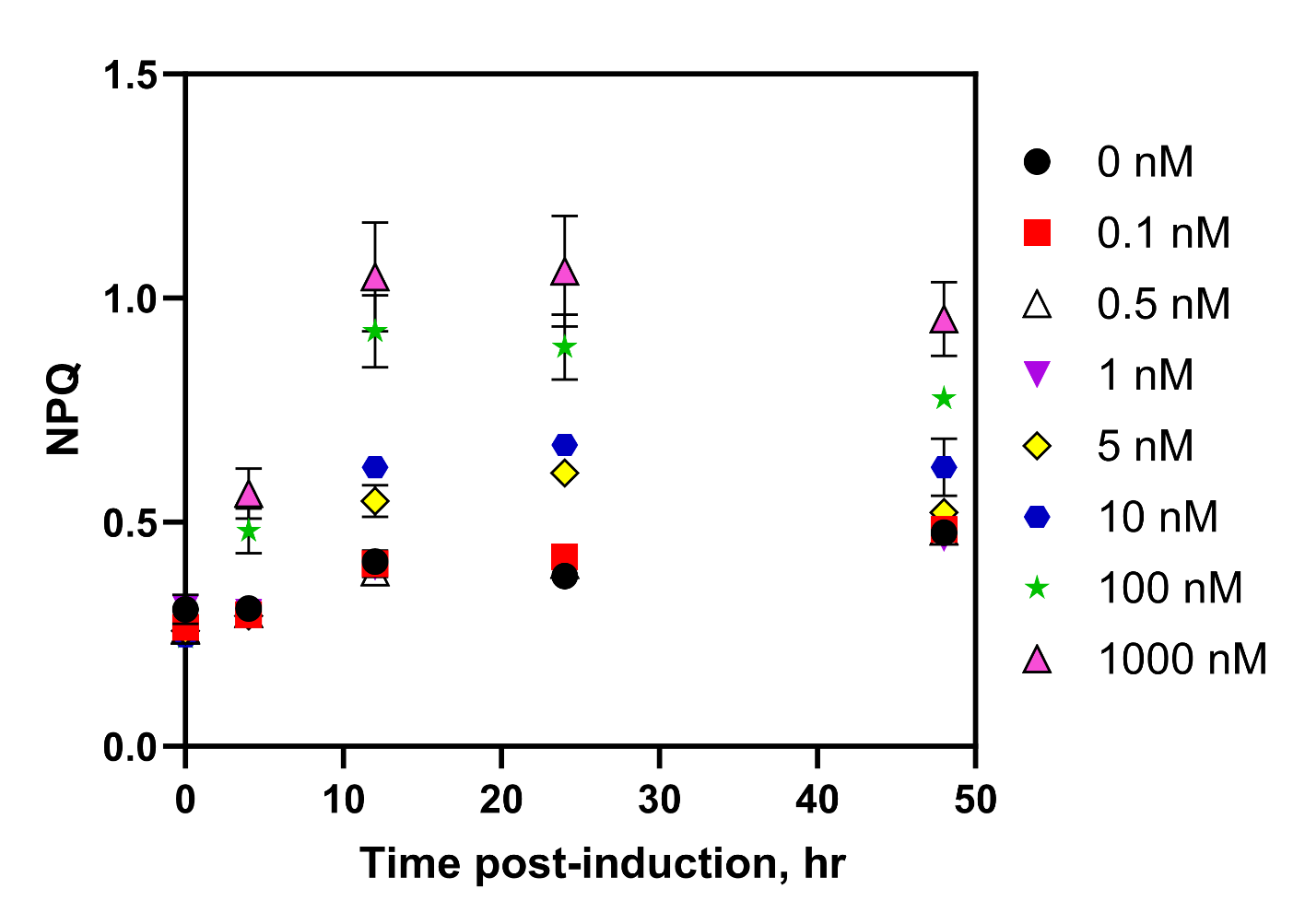


**Figure S6**

Tuning of NPQ capacity via chemical induction of ZEP3 expression. An inducible ZEP3 *Phaeodactylum* strain (*zep3*-2+iZEP3-6) was created via complementation of the *zep3*-2 mutant with a construct containing the native ZEP3 gene under the control of a β-estradiol-inducible synthetic promoter. The NPQ of the iZEP3 strain was measured with an IMAGING-PAM at different β-estradiol concentrations over the course of 48 hours. Cells were incubated and assayed in 24-well plates with 2 mL of f/2 medium to which was added 2 µL of β-estradiol at the needed concentration or 2 µL of ethanol as a vehicle control. Points are averages with error bars representing one standard deviation (n=3).
